# Mesoscopic Analysis of Fatigue Damage Development in Asphalt Mixture Based on Modified Burgers Contact Algorithm in Discrete Element Modeling

**DOI:** 10.3390/ma17092025

**Published:** 2024-04-26

**Authors:** Mingqiao Zhou, Wei Cao

**Affiliations:** School of Civil Engineering, Central South University, Changsha 410075, China; 214812265@csu.edu.cn

**Keywords:** indirect tensile fatigue, crack evolution, discrete element method, mesoscopic inspection, modified Burgers model

## Abstract

This study is aimed at examining the mesoscopic mechanical response and crack development characteristics of asphalt mixtures using the three-dimensional discrete element approach via particle flow code (PFC). The material is considered an assembly of three phases of aggregate, mortar, and voids, for which three types of contact are identified and described using a modified Burgers model allowing for bond failure and crack formation at contact. The laboratory splitting test is conducted to determine the contact parameters and to provide the basis for selecting three different load levels used in the indirect tensile fatigue test and simulation. The reliability of the simulation is verified by comparing the fatigue lives and dissipated energies against those from the test. Under cyclic loading, the internal tensile and compressive force chains vary dynamically as a response to the cyclic loading; both are initially concentrated beneath the top loading strip and then extend downward along the loading line. The compressive chains are oriented roughly vertically and develop an elliptic shape as damage grows, while the tensile chains are mostly horizontal and become denser. An analysis based on the histories of the numbers of different contact types indicates that damage mainly originates from bond failures among the aggregate particles and at the aggregate–mortar interfaces. In terms of location, cracking is initiated below the loading point (consistent with observations from the force chains) and propagates downward and laterally, leading to the macrocrack along the vertical diameter. The findings provide a mesoscopic understanding of the fatigue damage initiation and propagation in asphalt mixture.

## 1. Introduction

Considering the impacts of road construction and maintenance on the environment, it is of great significance to explore strategies that extend the service life of pavements and reduce resource consumption. Fatigue failure is one of the primary distress types of asphalt pavements due to the intricate impacts of traffic and environmental loading. So far, the fatigue characterization of asphalt mixtures is typically carried out in laboratories, and yet it is challenging to manage the material heterogeneity since they are multiphase composites [1,2,3]. Furthermore, it is difficult for laboratory experiments to capture the fatigue properties in the mesoscale, particularly with regard to the distribution and propagation patterns of the (micro)cracks, thereby posing challenges to fully characterizing the fatigue and fracture mechanisms [4,5]. It has been shown that the mechanistic characteristics and cracking mechanisms of asphalt mixtures may be effectively analyzed using numerical simulation techniques [6,7,8,9,10]. It is therefore of important significance to use virtual fatigue testing to study the fatigue cracking process from a mesoscopic perspective, which may provide potential insights for the durable and sustainable design of asphalt mixtures.

The two most common simulation approaches are the discrete element method and the finite element method. The finite element method is based on the mechanics theory of continuous medium, and its computational accuracy is significantly reduced or even fails to converge when simulating large deformations and cracking [11,12,13,14,15]. The discrete element method applies proper constitutive relationships at the particle contact to mimic the material’s complicated mechanical behavior, making it appropriate for simulating and analyzing heterogeneous, discontinuous, and massive deformation problems [16]. Utilizing the discrete element method to simulate the discontinuous materials of asphalt mixtures offers special benefits. The displacement of each discrete unit can be used as a basis for determining the degree of damage of the material structure under loading, the distribution of the contact force chains directly reflects the mesoscopic mechanical response, and the destruction of the internal contact bonds simulates the cracking behavior that occurs when the structure evolves to failure [17,18,19]. The PFC program is based on the discrete element method, which can simulate the cracking, particle separation, and motion behaviors of asphalt mixtures, and has been widely adopted in road engineering [20,21,22,23,24].

The PFC code is available in two dimensions (2D) and three dimensions (3D), and it is worth noting that it is challenging to describe the mesoscopic structure of asphalt mixtures using 2D DEM [25]. For instance, 2D DEM is unable to sufficiently account for aggregate interlocking and irregular aggregate shapes. The 3D models have advantages in these respects but the procedures of creating and optimizing them are computationally more expensive due to the complexity of interconnections and structures [26]. You et al. [27,28] developed 2D and 3D discrete element models to simulate the dynamic modulus test of asphalt mixtures and predict the dynamic modulus of asphalt mixtures using image processing and 3D digital generation technologies. They suggested that the DEM can predict the modulus change in a particular range of temperatures and loading frequencies and that the 3D model’s prediction results are superior. Wang et al. [16,29] used 2D and 3D discrete element methods, respectively, to perform virtual dynamic creep tests to study the creep mechanism of asphalt mixtures. Using a novel aggregate template technique, irregular polyhedrons were created and randomly produced to represent the coarse aggregate based on the probabilistic and Monte Carlo methods. Although it was discovered that both DEMs could accurately replicate dynamic creep testing, the 3D DEM approach yielded more reliable results.

The fatigue simulation of asphalt mixtures is a lengthy and complex process. More importantly, the DEM created by implementing the Burgers contact model in the PFC5.0 version of the software is unable to bear tensile force, and, therefore, the related studies in the literature are limited. Ma et al. [30,31] established a four-point bending beam fatigue simulation and used digital erosion modeling to assess the impact of various pore parameters on the fatigue life of asphalt mixtures. They also assessed the influence of void content, distribution, size, and direction on the fatigue life. Peng et al. [32] estimated the shear fatigue life of asphalt mixtures by simulating repeated uniaxial penetration tests using the 3D discrete element approach, which allowed for the examination of the shear fatigue performance as well as the identification of the affecting factors. Zhang et al. [33] simulated the low-temperature splitting behavior of asphalt mixtures using a linear bonding model to account for the viscoelasticity and evaluated the self-healing capability under fatigue loading. Peng et al. [34] introduced damage factors into the contact model to implement fatigue damage simulation and compared the mechanical responses of six asphalt layers under traffic loading. Pei et al. [35] used DEM to evaluate the damage performance of asphalt overlays on concrete pavement and assessed the effect of thermal fatigue stress on overlay cracking. The majority of existing studies are focused on the post-failure stage to examine the features of fatigue damage and the impacts of various influencing factors. The evolution characteristics and fracture process of asphalt mixtures throughout the fatigue failure have not been comprehensively investigated. In order to approximate the viscoelastic properties of asphalt mixtures, the linear contact model is widely used, or the Burgers model is considered.

**Figure 1:**
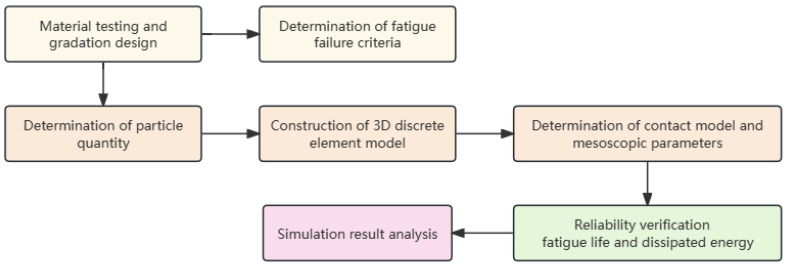
Research methodology flowchart.

This study utilizes the PFC3D software combined with the Burgers contact model to construct a 3D DEM of the indirect tensile fatigue test, in which the crack formation is simulated by means of contact bond damage. The Burgers contact model bonding algorithm is proposed, which can more accurately characterize the viscoelastic properties of asphalt mixtures in PFC software (version 5.0). The model verification is based on laboratory tests conducted at various stress ratio conditions. The mechanical behaviors of asphalt mixtures under cyclic loading and the progression of fatigue cracking are examined from a mesoscopic viewpoint, including the fatigue crack evolution law, aggregate displacement, and the formation law of tension and compression chains. The findings are expected to provide a theoretical basis for improving the understanding of fatigue damage mechanisms of asphalt mixtures. Figure 1 presents the flowchart of the overall methodology.

## 2. Materials and Methods

In this study, a #70 penetration-grade asphalt and basalt aggregate were selected in the laboratory testing, and the technical properties are shown in Table 1 and Table 2, respectively. The results of all raw materials met the requirements of the Technical Specification for Highway Asphalt Pavement Construction, JTG F40-2004 [36]. A dense-graded asphalt mixture with a nominal maximum aggregate size of 13 mm, AC-13, was selected for the fatigue characterization, and the aggregate gradation is shown in Table 3. The optimum asphalt content was determined to be 5.0% and the target void ratio was 4.0% according to the Marshall design method.

In order to select the appropriate fatigue loading levels, the splitting (indirect tensile fracture) test was carried out following the Standard Test Methods for Asphalt and Asphalt Mixtures in Highway Engineering, JTG E20-2011 [37]. The test was performed at a test temperature of 15 °C and a loading rate of 50 mm/min, using the Marshall samples at a dimension of Φ101.6 mm × 63.5 mm. The splitting strength was determined as

(1) $$R_{T}=\frac{2P_{T}}{\pi dh}$$

where *R_T_* is the splitting strength (MPa), *P_T_* is the peak load (N), *d* is the specimen diameter (mm), and *h* is the specimen height (mm).

**Figure 2:**
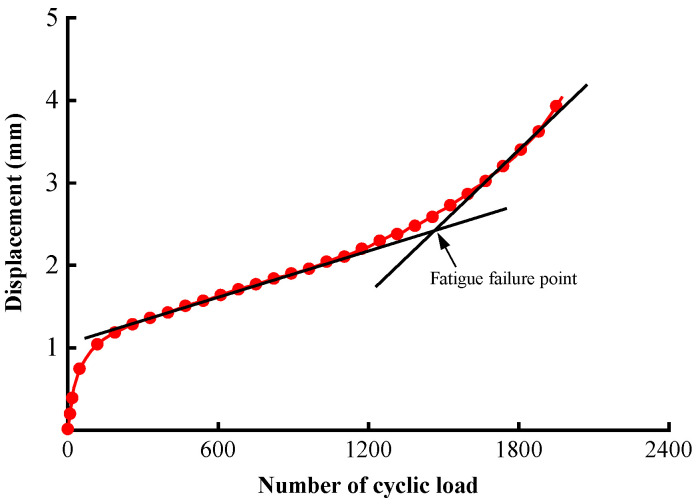
Definition of fatigue failure in the indirect tensile fatigue test.

Based on the splitting strength, the fatigue loading levels were selected through the stress ratio, i.e., the ratio of the stress amplitude induced by fatigue loading to the splitting strength. The indirect tensile fatigue test was conducted at the same temperature of 15 °C with three stress ratios of 0.2, 0.4, and 0.6 at a frequency of 10 Hz. Figure 2 illustrates a typical displacement versus load cycle curve obtained from the test. The inflection point dividing the stages of stable damage growth and accelerated growth is defined as the fatigue failure point in this study, and the corresponding number of cycles is the fatigue life.

## 3. Discrete Element Modeling

For the practical purpose of discrete element modeling, asphalt mixture can be thought of as a three-phase combination of voids, coarse aggregate, and asphalt mortar (consisting of asphalt binder, mineral filler, and fine aggregate). The coarse aggregate particles are set as irregularly formed blocks and the asphalt mortar particles as spheres, depending on the shape characteristics of the various components. The number of distinct particles should be ascertained before they are created. The interaction characteristics of the particles are then specified, followed by the establishment of the discrete element model of the test sample and the loading conditions.

### 3.1. Determining the Number of Particles

The mass–volume relationship of aggregates can be described by the gradation in 3D specimens to ascertain the number of discrete elements in each grain size interval [38]. The volume ratio of particles with varying particle sizes is determined by the asphalt–aggregate ratio, density, design gradation, and target void ratio, as indicated in Equation (2). Then, Equation (3) is used to determine the number of particles in each specified sieve size interval, and the obtained results are listed in Table 4.

(2) $$J_{D_{i}}=\frac{\left(P_{D_{i+1}}-P_{D_{i}}\right)}{100}\times \left(1-VV\right)/\left(1+\frac{a\rho_{c}}{\rho_{l}}\right)$$

(3) $$N_{D_{i}}=J_{D_{i}}\times V/\left(\frac{4}{3}\times \pi \times {\left(\frac{D_{i+1}+D_{i}}{2}\right)}^{3}\right)$$

where *D_i_* is the size of the *i*-th sieve; *J_Di_* is the volume fraction of the *i*-th coarse aggregate in the specimen; *P_Di_* is the passing ratio of coarse aggregate in the *i*-th sieve; *VV* is the design void ratio; *a* is the asphalt–aggregate ratio; *ρ_c_* is the aggregate density (kg/m^3^); *ρ_l_* is the density of the asphalt (kg/m^3^); *N_Di_* is the number of particles of the *i*-th coarse aggregate; and *V* is the total specimen volume.

### 3.2. Generating Discrete Unit Particles

In asphalt mixtures, the assortment of irregular aggregates with distinct properties makes up the load-bearing skeleton. The stresses, deformations, and cracking of asphalt mixtures are significantly influenced by the shape, geometric characteristics, and interlocking established between the aggregate particles. In this work, irregular particles were modeled using an aggregate template based on clumps; details are provided in the study of Dan et al. [39]. Realistic particle shapes and sizes of agglomerates were used to represent coarse aggregates (≥2.36 mm) in the clump-based model. Because pebbles in the same clump do not make contact, using clumps can significantly improve computational efficiency. A 3D scanner was used to create the digital aggregate templates for the discrete element simulation. After importing the template file into PFC3D, the bubble pack algorithm was employed to fill the entire template with pebbles [40]. The uniform-sized balls were used to represent the asphalt mortar as a continuous phase consisting of asphalt binder, filler, and fine aggregate.

**Figure 3:**
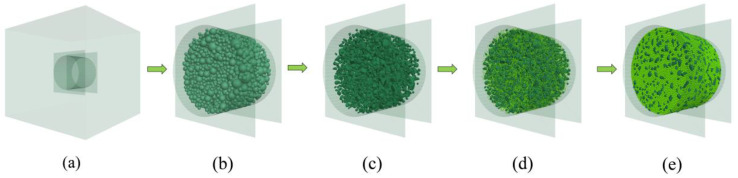
DEM building process: (**a**) the predefined model generation domain; (**b**) randomly generating aggregate particles based on gradation; (**c**) coarse aggregate particles generated by digital template; (**d**) generating asphalt mortar and the volume-reduction treatment; (**e**) gradually expanding the volume for model equilibrium.

A 3D aggregate generation program was developed, and a clump-based DEM of the AC-13 mixture was built to more accurately model the random distribution of aggregate. The generation process is depicted in Figure 3. As seen in Figure 3a, a cylindrical boundary (wall) of the same size as the actual specimen dimension was first created within a model-generating region that was preset in PFC3D. Ball-shaped particles were produced randomly throughout the cylinder, as depicted in Figure 3b, according to the particle numbers in Table 4. Afterwards, as seen in Figure 3c, all of the balls were swapped out for randomly numbered aggregate templates, and the resulting coarse aggregate particles were depicted as clumps. Subsequently, as shown in Figure 3d, balls with a radius of 1 mm representing asphalt mortar were used to fill the aggregate voids within the boundary. In the process of Figure 3d, the clumps and balls were first treated with a volume-reduction process, and then the particles were quickly dispersed using the gradual expansion method, which helped to achieve a well-balanced model. This treatment minimizes the overlap of discrete units within the model to quickly reach equilibrium [41]. The finally obtained DEM model is depicted in Figure 3e, with 3620 clumps representing the coarse aggregate and 45,421 balls representing the mortar.

### 3.3. Determining Contact Model and Mesoscopic Parameters

**Figure 4:**
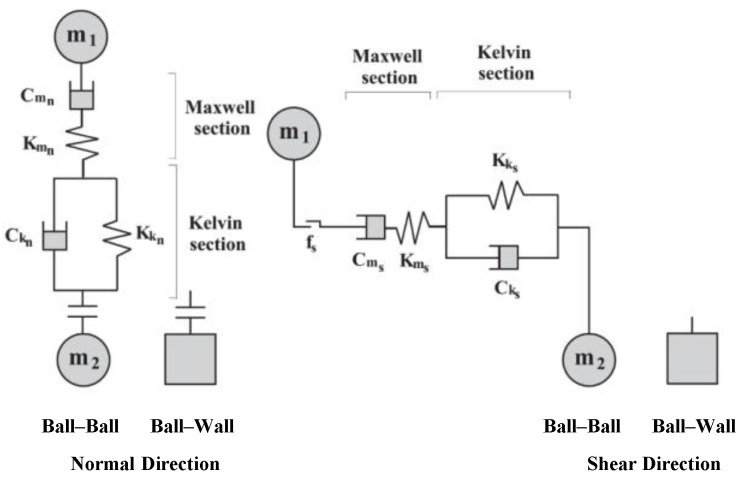
Burgers contact model embedded in PFC.

There are three different types of contact between the two types of particles: the contact between the particles of coarse aggregate, between the particles of asphalt mortar, and between the aggregate and mortar, as listed in Table 5. Given the viscoelastic nature of asphalt mortar, the Burgers model was adopted for contact involving this phase, so as to describe the time-dependent deformation behaviors of asphalt mixtures subjected to fatigue loading [42,43]. Nevertheless, the Burgers model built in the software is limited to only withstanding compressive loading in both normal and shear directions, and, therefore, by itself, it is unable to characterize the bond failure between particles. Given this inadequacy, recent studies have instead used the linear contact models [44,45]. Furthermore, when contact fails, the embedded Burgers model is unable to produce cracks, and damage sustained during loading is not visible due to internal mesoscopic changes in the model. In Figure 4, the Burgers contact model is displayed [46]. Therefore, the modified Burgers contact model approach was proposed in this study to remedy this deficiency by defining the mesoscopic mechanical modeling for contact involving the mortar.

**Figure 5:**
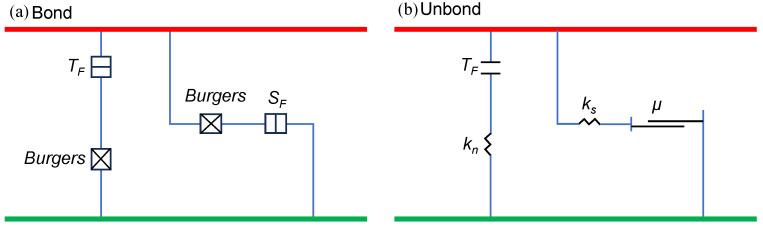
Modified Burgers contact model.

There are two cases in the modified Burgers contact model: bound and unbound, as seen in Figure 5. In addition, parameters for the tensile strength limit (*TF*) and shear strength limit (*SF*) are defined. The contact behavior always adheres to the Burgers model’s computational logic when the contact interface is bonded. Upon surpassing the strength limit, the contact link instantly breaks. A normal contact force (*F_n_*) and a tangential contact force (*F_s_*) can be formed by directionally breaking down the contact forces. The bond breaks in tension when the tensile strength limit is surpassed (*F_n_* > *TF*), and in shear when the shear strength limit is exceeded (*F_s_* > *SF*). The improved Burgers contact model reduces to a linear model when the contact breaks, as illustrated in Figure 5b, causing cracks to form at the contact center in the model.

The specific treatment of the modified Burgers contact model algorithm in PFC5.0 is as follows:

- Obtain a well-balanced DEM model in which the overlap between the particles was minimized;
- Set all contact forces to zero and the computation of the mesoscopic contact model to the incremental mode;
- Execute the *ball fix* and *clump fix* commands to fix all particles, iterate over all contact with the asphalt mortar, and set the contact to a hard activated state using the *contact.activate* function;
- Execute the *solve* command to dissipate the internal stresses of the model;
- Execute the *free* command to allow the particles to move freely.

Following the aforementioned process, bonds that can withstand tensile forces were produced via the modified Burgers contact model. During the computation, once either tensile or shear damage occurred at a contact, the program immediately detected it and produced a crack at this location and, hence, the contact failure.

**Figure 6:**
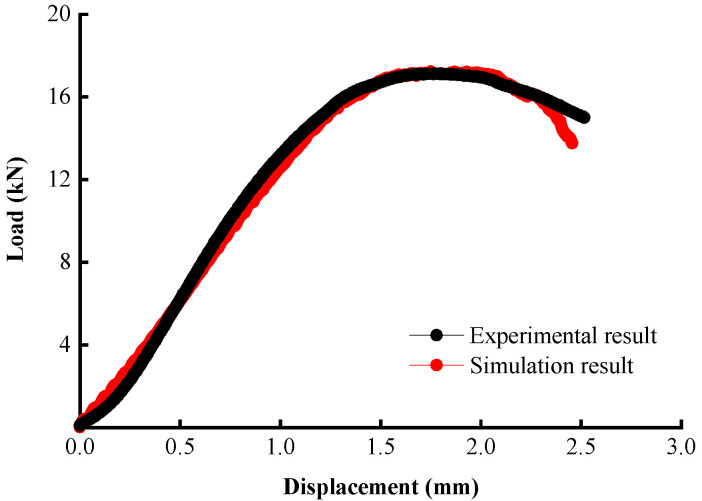
Comparison of the load–displacement curves between the test and simulation.

In discrete element simulation, mesoscopic parameters generally cannot be obtained in a straightforward manner but are converted from macroscopic parameters that are determined based on experiments. According to the PFC3D manual [47], the macroscopic parameters of the Burgers model can be converted into the normal and tangential contact parameters of the Burgers contact model. According to the DSR test results of asphalt mastic by Nian et al. [48], the mesoscopic parameters of the modified Burgers contact model were calculated. The tensile strength limit *TF* was determined with the splitting test. A relatively high value of the shear strength limit *SF* was selected in this investigation because asphalt mixtures primarily experience tensile failure in the indirect tensile fatigue test. A trial-and-error approach was followed to calibrate the model parameters using the splitting test curve, and the results are shown in Table 6 and Table 7. Figure 6 illustrates the comparison of the load–displacement curves from the test and from the simulation; an overall excellent agreement was noted.

### 3.4. Loading and Boundary Conditions

The same loading and boundary conditions were attempted for both the test and simulation for consistency. The 12.7 mm wide loading strips at the top and bottom were described using rigid walls of the same size; the bottom strip was fixed while the top strip was subjected to compressive stress in the load-controlled mode. Specifically, in the indirect tensile fatigue simulation, a haversine stress waveform with a frequency of 10 Hz was utilized. The stress amplitudes were determined with the preset stress ratios, and a minimum stress equal to 5% of the amplitude in each case was applied to maintain contact between the strip and specimen throughout the simulation.

## 4. Results

### 4.1. Verification of Fatigue Simulation

**Figure 7:**
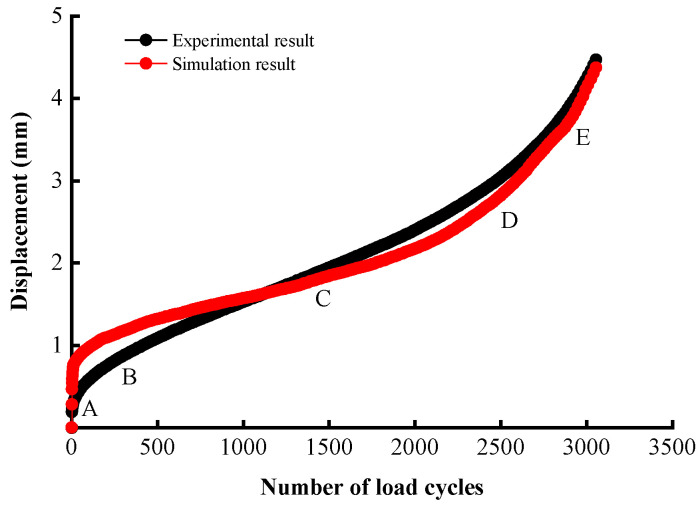
Comparison of displacement–cycle curves (stress ratio 0.4).

The reliability of the numerical simulation was examined by comparing the fatigue lives and displacement–cycle curves from the simulation against those from the test at different stress ratios. In order to track the displacement of the loading point, a *Fish* script was developed, and the resulting comparisons are shown in Table 8 and Figure 7.

As shown in Table 8, higher loading levels resulted in shorter fatigue lives, as expected. Increasing the stress ratio from 0.2 to 0.4 and from 0.4 to 0.6 results in 68.55% and 88.92% decreases in fatigue life, respectively. Also, the relative errors in fatigue life are low for all stress ratio conditions. Figure 7 shows a good agreement between the displacement versus load cycle curves at the stress ratio of 0.4. Both present the typical S-shaped histories that are characterized by three stages: A to B, the compression stage with rapid deformation at the start of loading; B to D, the steady stage of crack development and deformation accumulation; and D to E, the failure stage exhibited as rapid growth of deformation.

**Figure 8:**
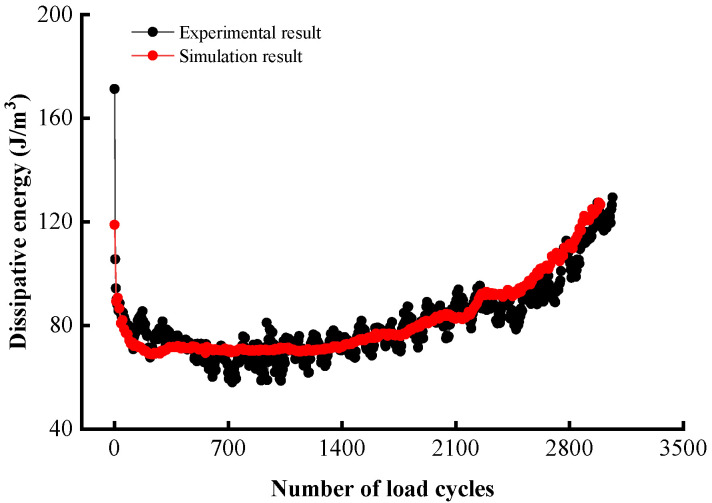
History of the dissipated energy (stress ratio 0.4).

The stress–strain relationship of asphalt mixtures under cyclic loading is shown as a hysteresis curve, by which the enclosed region represents the energy dissipated during a cycle. Asphalt mixtures with high dissipative energy can absorb and dissipate more energy when loaded, reducing energy return to the asphalt structure, thereby reducing fatigue damage [49]. To further confirm the simulation’s accuracy, the dissipated energy per cycle on average and cumulative dissipated energy were calculated up to the failure point using the stress–strain curves, and the results are given in Figure 8 and Table 9.

Figure 8 shows the experimental and simulated dissipated energy histories for the stress ratio of 0.4, and an overall excellent agreement was noted between the two. The material first experienced a sharp decline in the dissipated energy, and then entered a relatively stable plateau phase for load cycles between 50 and 2500. Afterwards, the dissipated energy started to rise rapidly and this accelerated energy dissipation signifies material failure. Table 9 compares the dissipated energy results from the test and simulation and indicates a good agreement in all stress ratio scenarios; the relative error is mostly below 10%. There are clear trends that with the rising stress ratio, the cumulative dissipation energy decreases while the average dissipated energy increases. This observation is expected as higher stress levels would produce more damage in a cycle and, thus, more energy dissipated per cycle on average, whereas the fatigue life is substantially reduced as seen in Table 8, and, hence, the lower cumulative energy. The comparison results based on fatigue life and dissipated energy validated the reliability and accuracy of the discrete element model and the virtual splitting and fatigue tests.

### 4.2. Contact Analysis

#### 4.2.1. Evolution of Force Chains

**Figure 9:**
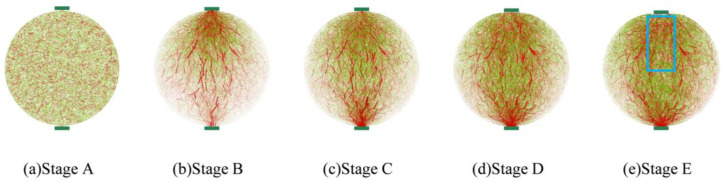
Distribution of force chains at different damage stages.

Under the continuous action of cyclic loads, the internal contact forces in the discrete element model undergo dynamic changes accordingly. The force chain from the virtual specimen was extracted to analyze the mechanical responses during damage evolution from the mesoscopic viewpoint. Figure 9 shows the transmission path and development of the contact force chains in the specimen at different damage states as indicated earlier in Figure 7. In Figure 9, the red columns represent compressive contact, and the green columns for tensile, while the column thickness reflects and is proportional to the magnitude of the contact force.

Stage A indicates the initial equilibrium state prior to the load application; the tensile and compressive contacts are randomly distributed across the model. After a limited number of cycles to reach stage B, primary compressive chains emerge around the top and bottom loading strips, indicating the transmission of the compressive force between the two strips. Meanwhile, the tensile contacts are mainly present in the upper half of the specimen, i.e., close to the loading point (noting that the bottom strip is fixed). As the cyclic loading continues from stages B to D, both the number and magnitudes of the compressive as well as tensile contacts increase progressively in the vicinity of the bottom strip. Moreover, the tensile contacts are mainly concentrated along the vertical line connecting the two strips, indicating the initiation and accumulation of damage in this region. At stage E, where the material loses the fatigue resistance, the tension chains inside the indicated rectangular area in Figure 9e become sparse due to the failure of tensile bonds and, hence, the appearance of macrocracks, which is accompanied by the depression of the top loading strip. These findings are in good agreement with general experimental observations preceding the sample failure in the indirect tensile fatigue test.

Under the external cyclic loading, the distribution patterns of the tensile and compressive force chains also exhibit alterations accordingly. At low-stress or -damage levels, the compressive force chains are roughly distributed vertically from the top to the bottom strip. At high-stress or -damage levels, the pattern changes into an elliptic shape linking the strips. The overall distribution of tensile force chains gradually moves down during the repeated loading, which suggests that in the indirect tensile loading mode, the damage initiates at the top loading point and then propagates downward to finally fracture the specimen.

#### 4.2.2. Evolution of Contact Orientations

**Figure 10:**
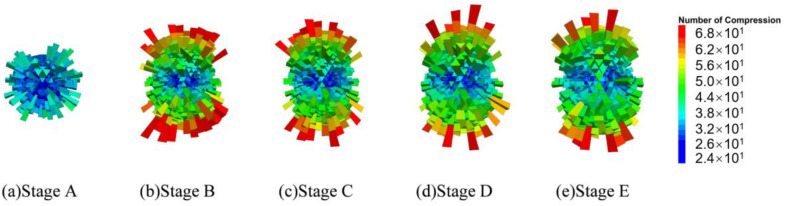
A 3D fabric diagram of compressive contacts.

**Figure 11:**
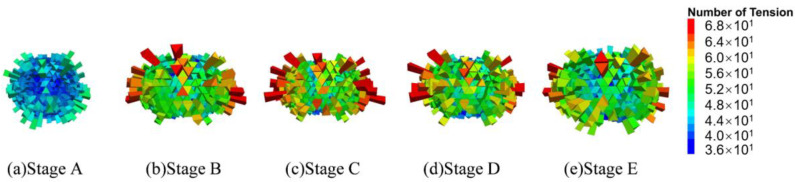
A 3D fabric diagram of tensile contacts.

Following an extensive investigation of the model’s contact structure, statistical findings about the number of tensile and compressive contacts in every direction inside the virtual specimen were obtained, and the results are presented as 3D fabric diagrams in Figure 10 and Figure 11. The length of a fabric unit represents the number of contacts in its direction.

As seen in Figure 10, the majority of the compressive contacts are found to be orientated less than 90 degrees from the *z*-axis (vertical). The number of fabric units gradually increases in the vertical direction from stages A to C, activating more compressive connections to carry the load. On the other hand, as the compressive chain evolves into the elliptic pattern from stages C to E, the number of long fabric units gradually decreases and their directions deviate from the *z*-axis. Results of the tensile contacts given in Figure 11 present a sharp contrast to the compressive ones, as the fabric components are mostly arranged along the horizontal direction from stages B to E. The Poisson effect is responsible for this expected phenomenon. Moreover, the number of long fabric units decreases from stages C to E, indicating a progressive loss of tensile bonds and, thus, the increasing degree of damage as the specimen approaches failure.

#### 4.2.3. Slice Analysis of Contact

**Figure 12:**
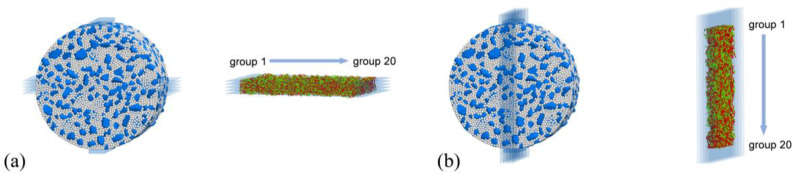
Extracted contact slices: (**a**) horizontal direction and (**b**) vertical direction.

The above investigation suggests that the horizontal and vertical planes passing through the specimen center are of the most physical significance in terms of load transmission and damage initiation/accumulation. Further details in these two regions can be revealed by extracting the specific contact information through a slice analysis. The following procedure was developed for this purpose. A *Fish* script was prepared to create five cutting planes with a 2 mm gap between one another along each of the horizontal and vertical diameters. The obtained slices in each direction were further divided evenly into 20 groups, as illustrated in Figure 12. The results of the groups from all slices having the same horizontal or vertical locations were then averaged to reduce the numerical random errors.

**Figure 13:**
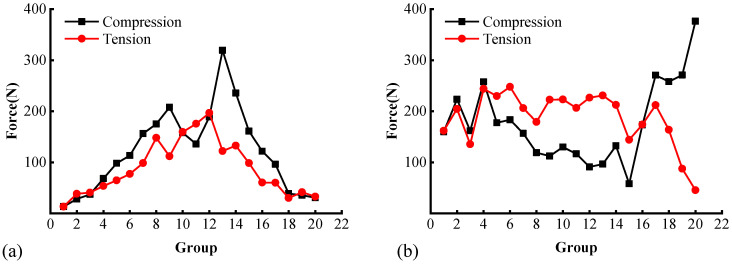
Compressive and tensile contact force profiles: (**a**) horizontal direction and (**b**) vertical direction.

Figure 13 shows the average magnitudes of compressive and tensile contact forces along the two directions. In Figure 13a, the profile of the compressive force shows two peaks of 207.68 N and 319.58 N at groups 9 and 13, respectively. A closer inspection indicates that these two locations are at the intersection of the elliptic path of the compressive force chain with the horizontal diameter; see Figure 9. In contrast, the tensile profile exhibits only a single peak of 196.9 N around the specimen center at group 12, close to the fracture path. Both the compressive and tensile profiles attenuate to zero towards the specimen edge.

Figure 13b presents the force distributions along the vertical diameter. The compressive contact force first decreases and then increases along the specimen height. Specifically, the average magnitude for contact groups 2–4 and 17–20 are 214.4 N and 294.1 N, respectively, which are comparable to the two peaks of the compressive contacts in Figure 13a. The force magnitude at the specimen center is substantially lower than the edge, which means that the particles near the two loading strips are subjected to higher pressures. The tensile profile exhibits a slightly lower fluctuation compared to the compressive contacts. Before reaching the bottom loading strip, the tensile force varies in a moderate range around 200 N, a level comparable to the peak tensile force in the horizontal direction as seen in Figure 13a. Note that the drop in the tensile force at group 15 is attributed to the presence of coarse aggregate particles with a grain size greater than 13.2 mm at this location. Therefore, the space considered therein is occupied by a clump without contact, and, hence, the sudden decrease.

### 4.3. Particle Displacement and Contact Quantity

**Figure 14:**
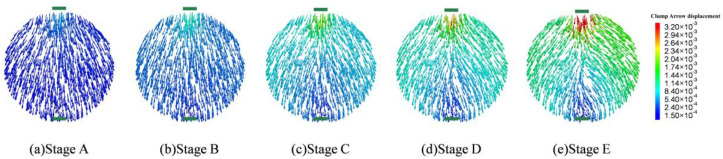
Displacement contour of aggregate particles at different stages.

The particle interactions form the aggregate skeleton of asphalt mixtures, which necessitates the analysis of the coarse aggregate’s movement during indirect tensile fatigue simulation. Figure 14 provides the displacement contour of coarse aggregate particles at different stages. Due to symmetry in the specimen geometry as well as the loading and boundary conditions, the displacement contour is overall symmetric with respect to the vertical loading line at different damage states. The aggregate particles move collectively downward during the first loading stage (stage A), with a maximum displacement of 0.591 mm just beneath the loading strip. The particles then move symmetrically to the left and right from stages B to D, with a total horizontal displacement of around 2 mm, suggesting that the asphalt mortar as a bonding agent is subject to tension along the vertical diameter of the specimen, thereby counteracting the aggregate’s tendency of lateral movement. Upon reaching stage E, the particles have shifted considerably by a maximum horizontal displacement of 5.87 mm, in contrast to the small downward movement of particles beneath the top loading strip. The significant lateral expansion at this late stage is caused by the macrocracks developed along the loading line, signifying material failure.

**Figure 15:**
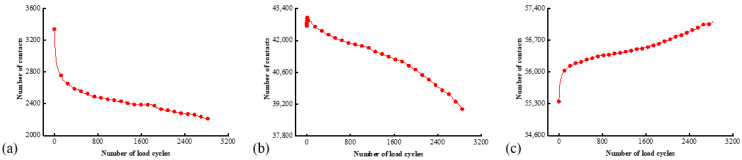
The numbers of different contact types: (**a**) between aggregate particles; (**b**) between aggregate and asphalt mortar; and (**c**) between asphalt mortar.

The locations of aggregate and asphalt mortar particles are constantly moving during the cyclic loading, thereby affecting the number of different contact types and the internal aggregate structure. Figure 15 provides the histories of a number of different contacts extracted from the fatigue simulation. The quantity of contacts between aggregate particles constantly drops during the repeated loading as shown in Figure 15a, which can be attributed to the progressive disintegration of the specimen and, thus, the reduced inter-aggregate contacts as damage accumulates. According to Figure 15b, the reduction in the number of contacts between aggregate and mortar particles is more pronounced. It is suggested that the aggregate–mortar contact is more sensitive to the particle movement inside the asphalt mixture. A slight increase in the number of contacts by 436 is also noted during the initial few cycles, which can be ascribed to the densification effect mainly owing to the movement of the mortar particles. The number of interactions within the mortar phase exhibits an increasing trend as seen in Figure 15c. These observations suggest that in the indirect tensile loading mode, the contacts between aggregate particles and at the aggregate–mortar interface are the weak points more prone to bond failure. Damage is less likely to develop within the continuous phase of asphalt mortar.

### 4.4. Evolution of Cracking Damage

**Figure 16:**
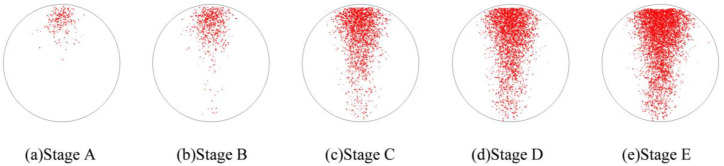
The process of crack initiation and propagation in simulation.

In this study, the initiation and development of internal cracks were investigated through the evolving distribution of contact failures in the asphalt mixture specimen. The results are shown in Figure 16, in which the cracks are represented by red disks. Upon a few load cycles at stage A, a small number of cracks appear in the vicinity of the loading point. The number of cracks increases and also extends downward as the loading continues to stage B. From stages C to E, the cracks are primarily centered along the vertical diameter of the specimen, with a denser and wider distribution towards the top loading strip. The crack distribution at stage E close to fatigue failure indicates the specimen depression and macrocrack initiation at the loading point, in agreement with the previous observations based on Figure 9 and Figure 14. This asymmetric pattern of damage was considered as a result of the viscoelastic (delayed elastic) nature of asphalt mixtures, despite the symmetric loading and boundary conditions. The bottom strip was reactively acting onto the specimen and, thus, the damage initiation and development therein lagged behind those beneath the top strip.

**Figure 17:**
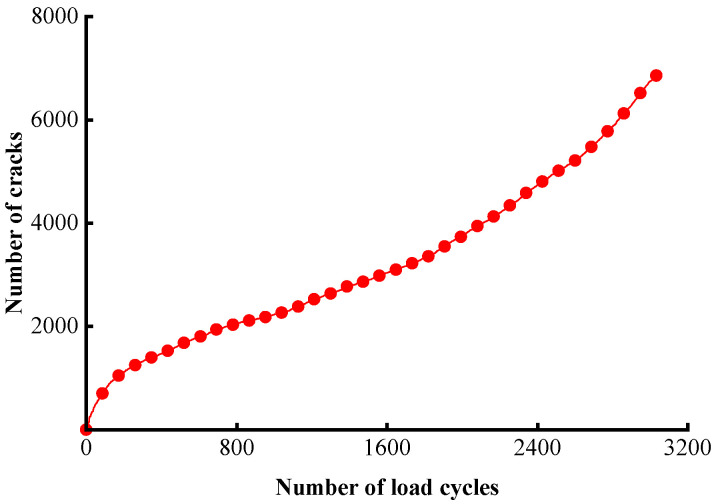
The history of the number of cracks.

The total number of cracks was also tracked in real time during the simulation, as illustrated in Figure 17. The overall increasing trend featuring three segments and the S-shape is analogous to the displacement history given earlier in Figure 7. A total of 4362 cracks are developed up to the failure point. The damage accumulates rapidly below 200 load cycles, yielding a number of cracks accounting for 25.5% of the total number. The material then enters a steady stage of damage growth before reaching the final acceleration phase around 2400 load cycles.

### 4.5. Mesoscopic Analysis of Model Slices

**Figure 18:**
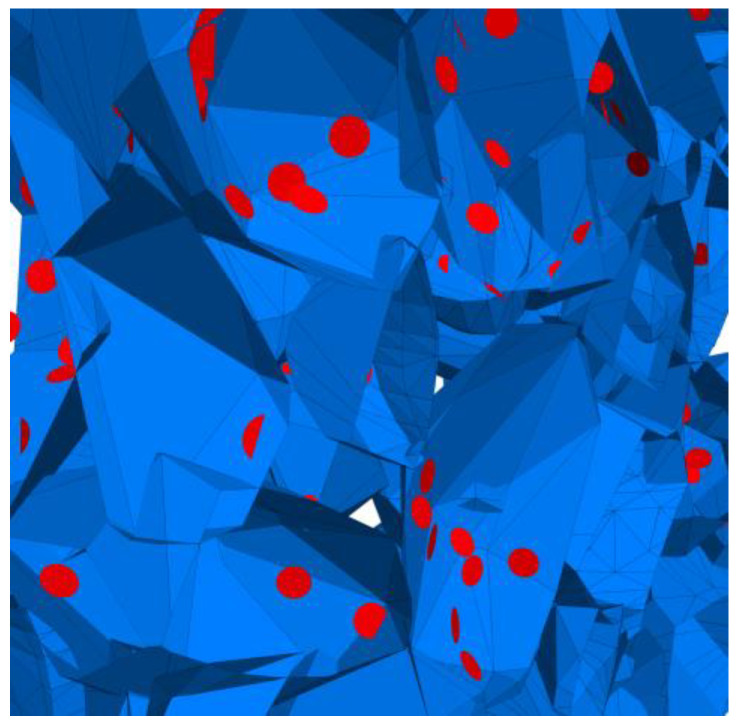
Details of crack distribution in the 3D model.

Table 10 shows the front and side views of the middle cross-section of the virtual specimen at different loading stages. The cracks represented as red disks appear on and around the aggregate due to projection but are actually located at the aggregate–mortar interfaces in the 3D model. The specific details are illustrated in Figure 18. Almost no cracks are identified between the mortar balls. The deformation at the loading point is moderate prior to the appearance of the macrocrack, but afterwards, from stages D to E, the macrocrack grows fast, thereby causing an acceleration in the model’s overall deformation. Up to the final stage, the relative positions of the particles did not alter significantly, but the overall structure was separated into two halves. The upper portion of the specimen exhibits more dramatic deformation according to the side view; further, the aggregate and mortar particles at the top margin are arranged in a looser structure with greater gaps between the particles. The reason is the high number of cracks beneath the loading strip and, thus, less bonding between the particles (Figure 16), resulting in a lower strength of the upper portion and consequently a higher susceptibility to deformation.

## 5. Conclusions

- This study uses the three-dimensional discrete element approach to numerically investigate the fatigue failure process of asphalt mixtures. The indirect tensile fatigue loading mode is selected to assess the mechanical responses and damage development characteristics under repeated loading. The key findings are as follows:
- A good agreement between the laboratory fatigue test and simulation results is obtained; the relative error of simulation in terms of fatigue life and dissipated energy is within 10%. The modified Burgers contact model provides a satisfactory characterization of the macroscopic behavior of asphalt mixtures.
- The internal force chains vary dynamically as a response to the applied cyclic loading. The compressive chains are roughly oriented vertically and are the main carriers of the external load; they are initially concentrated along the vertical diameter, and then develop into an elliptical pattern as the number of load cycles increases. The tensile chains are initiated beneath the top loading strip and then propagate downward mainly along the vertical loading line; they are oriented horizontally and become denser as damage accumulates.
- The displacement contour of aggregate particles presents a symmetric pattern in which the particles move downward and laterally. The lateral expansion becomes more significant when approaching the fatigue failure point. Damage mainly originates from the failure of bonding among the aggregate particles and at the aggregate–mortar interfaces as suggested by the reducing numbers of the two contact types.
- In the indirect tensile fatigue simulation, cracks are initiated below the loading point due to the depression of the top strip, and then extend downward, forming macrocracks along the vertical diameter. This observation is in agreement with typical experimental findings and highlights the complication due to material viscoelasticity and boundary effects in this loading mode.

By simulating the fatigue damage process of asphalt mixtures, the initiation and development mechanisms can be better understood. This would ultimately help reveal the performance degradation path of asphalt mixtures in pavement structures and assist in optimizing the mix design, material selection, and structural design. Discrete element simulations can also supplement experimental studies and be used to understand/validate phenomena observed in the laboratory.

Nevertheless, certain limitations in the present work should be acknowledged. For instance, aggregate particles may fracture under high stress, but this phenomenon was not considered. Both the fatigue testing and simulation were limited to relatively low numbers of load repetitions, and the results are not expected to yield reasonable extrapolation to high cycle fatigue. Additionally, this study only performed simulation analysis on indirect tensile fatigue tests. In the future, more fatigue simulations using semi-circular and bending beam configurations, for instance, can be carried out to comprehensively explore the fatigue damage behavior of asphalt mixtures.

## Figures and Tables

**Table 1:** Technical properties of the asphalt binder.

Properties	Results	Technical Requirements
Penetration (25 °C, 100 g, 5 s)/0.1 mm	67.8	60–80
Softening point (Ring ball)/°C	47	≥46
Ductility (15 °C, 5 cm/min)/cm	150	≥100
Dynamic viscosity (60 °C)/Pa·s	224	≥180

**Table 2:** Technical properties of the aggregate.

Properties	Sieve Size (mm)		Technical Requirements
	2.36~9.5	9.5~16	
Apparent relative density	2.741	2.841	≥2.50
Crushing value (%)	/	11.1	≤28
Water absorption rate (%)	1.2	0.9	≤3.0
Needle flake content (%)	/	12.8	≤18

**Table 3:** Aggregate gradation.

Sieve size (mm)	16	13.2	9.5	4.75	2.36	1.18	0.6	0.3	0.15	0.075
Passing ratio (%)	100	97.8	77.4	50.9	35.3	23.9	18.1	13.2	9.9	7.7

**Table 4:** Number of particles for each size in 3D specimen.

Particle size/mm	2.36~4.75	4.75~9.5	9.5~13.2	13.2~16
Number of particles	2856	603	115	46
Volume/cm^3^	67	114	88	9

**Table 5:** Contact models.

Contact Type	Selected Contact Models
Between coarse aggregate particles	Linear model
Between asphalt mortar particles	Modified Burgers contact model
Between coarse aggregate and mortar particles	Modified Burgers contact model

**Table 6:** Linear contact model parameters.

*E* (GPa)	*k_n_* (N/m)	*k_s_* (N/m)	*v*	*μ*
55.5	1.11 × 10^8^	8.22 × 10^7^	0.35	0.5

**Table 7:** Burgers contact model parameters.

*K_mn_* (Pa·m)	*K_kn_* (Pa·m)	*K_ms_* (Pa·m)	*K_ks_* (Pa·m)	*C_mn_* (Pa·m·s)	*C_kn_* (Pa·m·s)	*C_ms_* (Pa·m·s)	*C_ks_* (Pa·m·s)	*T_F_* (N)	*S_F_* (N)
1.47 × 10^6^	2.96 × 10^5^	5.89 × 10^5^	1.19 × 10^5^	2.48 × 10^6^	1.79 × 10^6^	9.90 × 10^5^	7.71 × 10^5^	34.1	60.0

**Table 8:** Comparison of fatigue lives.

Stress Ratios	Test	Simulation	Error
0.2	8122	8569	5.5%
0.4	2473	2260	8.6%
0.6	274	301	9.8%

**Table 9:** Dissipated energy results from fatigue tests and simulations.

Stress Ratios	Cumulative Dissipated Energy (J/m^3^)		Average Dissipated Energy (J/m^3^)		Error
	Test	Simulation	Test	Simulation	
0.2	253,409.6	270,134.3	/	/	6.6%
	/	/	31.2	31.5	1.0%
0.4	187,362.6	173,666.4	/	/	7.3%
	/	/	75.8	76.8	1.4%
0.6	67,236.8	74,767.3	/	/	11.2%
	/	/	245.4	248.4	1.2%

**Table 10:** Cross-sectional views of the virtual specimen.

Stage	Front	Side
Stage A	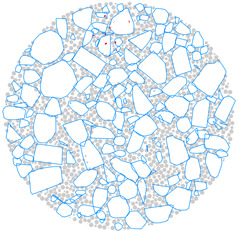	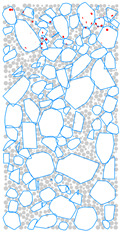
Stage B	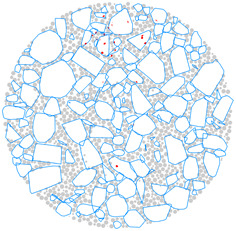	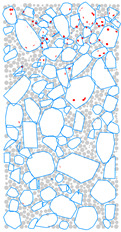
Stage C	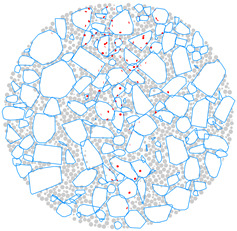	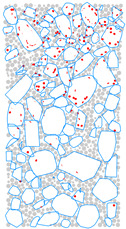
Stage D	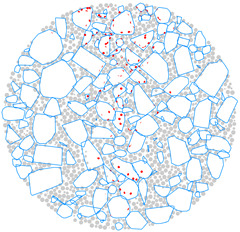	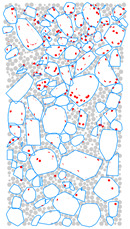
Stage E	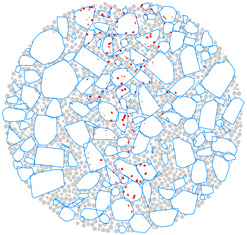	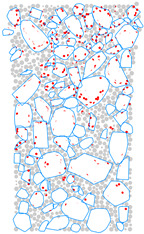

Note: the red disks in the slice represent cracks.

## Data Availability

The data will be available upon reasonable request to the corresponding author.
